# Adjustments in Shoulder and Back Kinematics during Repetitive Palletizing Tasks

**DOI:** 10.3390/s22155655

**Published:** 2022-07-28

**Authors:** Saeb R. Lamooki, Lora A. Cavuoto, Jiyeon Kang

**Affiliations:** 1 Mechanical and Aerospace Engineering, University at Buffalo, Buffalo, NY 14260, USA; saebraga@buffalo.edu; 2 Industrial and Systems Engineering, University at Buffalo, Buffalo, NY 14260, USA

**Keywords:** upper-body kinematics, repetitive task performance, back bending, loading and unloading, ergonomics

## Abstract

Repetitive task performance is a leading cause of musculoskeletal injuries among order-picking workers in warehouses. The repetition of lifting tasks increases the risk of back and shoulder injuries among these workers. While lifting in this industry is composed of loaded and unloaded picking and placing, the existing literature does not address the separate analysis of the biomechanics of the back and shoulder for these events. To that end, we investigated the kinematics of the back and shoulder movements of nine healthy male participants who performed three sessions of a simulated de/palletization task. Their back and shoulder kinematics were sensed using an optical motion capture system to determine the back inclination and shoulder flexion. Comparison of the kinematics between the first and last sessions indicated statistically significant changes in the timings, angles, coordination between the back and shoulder, and moments around the shoulder (p<0.05). The majority of the significant changes were observed during the loaded events, which confirms the importance of the separation of these events for biomechanical analysis. This finding suggests that focusing worker evaluation on the loaded periods can provide important information to detect kinematic changes that may affect musculoskeletal injury risk.

## 1. Introduction

Musculoskeletal disorders (MSDs) are responsible for approximately USD 50 billion in annual costs in the U.S. alone [1]. In 2020, the incidence rate for nonfatal occupational injuries and illnesses involving days away from work for MSDs was at 25.4 per 10,000 full-time workers, and the injured workers needed a median of 14 days before returning to the work [2]. Lifting, climbing, pushing, pulling, and pivoting, which are the primary movements in MMH activities, pose great risk of injury on the back and shoulder [3]. Particularly, order-picking activities in warehouses and distribution centers require a significant amount of manual handling. It is estimated that about 75% of warehouse tasks and 50% of warehouse operation cost are associated with order picking [4]. Some locations achieve up to 1000 picks per person hour [5]. It is the most labor-intensive process in manual warehouses and the most expensive procedure in automated warehouses [5].

Picking and placing, which are the main tasks in the order-picking occupations, are made up of two major movements of back and shoulder flexion. The most prevalent MSD among MMH workers is lower back pain (LBP) [6] where lifting, carrying loads, and frequent bending are the most consistently reported risk factors [7,8,9]. A longer duration of back flexion in mild (20° to 40°) and severe (>45°) ranges have been found to be associated with LBP [10]. Furthermore, Norman et al. [11] reported that extreme back flexion (>60°) increases the risk of LBP. Shoulder injuries are similarly prevalent among workers. Non-neutral shoulder postures during occupational tasks are found to be associated with shoulder injuries and chronic musculoskeletal pain [12,13]. Punnett et al. [14] reported that shoulder pain in automobile assembly workers was associated with extreme shoulder flexion or abduction. Additionally, motor variability (MV) has been shown to be a potential risk factor for work-related MSDs [15,16]. It has been hypothesized that increased MV can be associated with reduced risk of injury [17,18,19] based on findings that suggest reduced MV is observed in less experienced workers [20] as well as in workers with pain [21,22]. To that end, kinematic investigation of prolonged repetitive task performance that relies heavily on the back and shoulder movements remains critical to addressing the adverse impact of occupational MSDs.

To that end, the effect of material handling in distribution centers and warehouses has been investigated in terms of back and shoulder biomechanics [23,24,25,26,27,28]. Gallagher and Heberger [29] have investigated the biomechanical demands on the lower back during the lifting and lowering of bags in mining commodity warehouses for different pallet layouts. Moreover, Jorgensen et al. [30] explored the effect of pallet distance from the storage site on worker biomechanics to evaluate the risk of development of lower back disorders. The effect of material handling has also been investigated on shoulder biomechanics in warehouse settings [31]. The effect of prolonged repetitions of lifting has been investigated on workers’ biomechanical responses [32,33]. However, the majority of previously published studies have investigated the lifting task as a single activity and evaluated the back and shoulder kinematics during this single event. The bending and straightening during lifting and lowering are two separate actions that can be affected differently by repetition and fatigue. Moreover, as palletization in warehouses is composed of pickings and placings, the bending and straightening during picking and placing are divided into loaded and unloaded events and need to be assessed separately.

Therefore, in the present study, we investigated the effect of a repetitive palletizing/depalletizing task on the kinematics of the back and shoulder during the bending and straightening of picking and placing. We evaluated the back and shoulder flexion in terms of timing, angles, coordination, movement variations, and moments in the search for potential compensatory kinematic strategies.

## 2. Methods

Nine young male adults participated in this pilot study with the ages of 28.1 ± 3.2 years, weight of 75.1 ± 10.4 kg, height of 176.8 ± 7.9 cm, and BMI of 23.9 ± 2.4 kg/m^2^ (average ± standard deviation). The participants were not skilled industrial workers and they were novice to the palatalizing task.The participants were healthy with no history of musculoskeletal disorders, which was self-reported. The experimental protocol was approved by the University at Buffalo’s institutional review board and all participants provided written informed consent prior to the experiments.

### 2.1. Experiment Design

**Figure 1:**
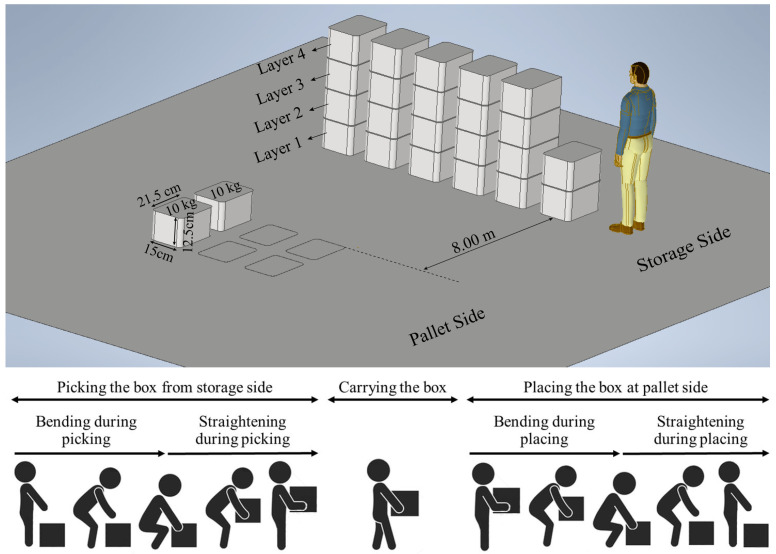
The (**upper**) panel shows the schematic of the experiment area with the pallet and storage locations. The distance between two locations were 8 m apart and each box weighed 10 kg. The (**lower**) panel shows the sequence of bending and straightening during picking and placing at the storage and pallet sides.

In this study, we simulated a manual material handling/order picking task, performed at warehousing and distribution centers as shown in Figure 1. The task included picking up a box at a storage location, carrying the box to a pallet, and loading the box on the pallet before returning to the storage location. Participants moved a total of 24 boxes, each weighing 10 kg. A metronome was used to control the task pace such that picking the box, carrying the box between the storage and pallet locations (8 m apart), and placing the box lasted for 12 s. This frequency of 5 picks/min was slightly higher than a typical average workplace pick rate of 180 picks per person per hour. However, all participants were able to complete the task without being rushed. The metronome was used to support consistent task demands across the test period and across participants. Once the pallet was fully loaded, the participant moved the boxes to their original location at the storage side with the same pace, reversing the loading process. The entire loading/unloading session lasted for approximately 10 min to move the 24 boxes. Each participant completed 3 sessions of palletizing/depalletizing with a 10 min break between the sessions. The top panel in Figure 1 presents the schematic of the experimental area as well as the area and the box dimensions. Moreover, the bottom panel in Figure 1 shows the sequence of bending and straightening during picking a box from the storage side and placing at the pallet side at ground level (layer 1).

### 2.2. Instrumentation and Data Acquisition

**Figure 2:**
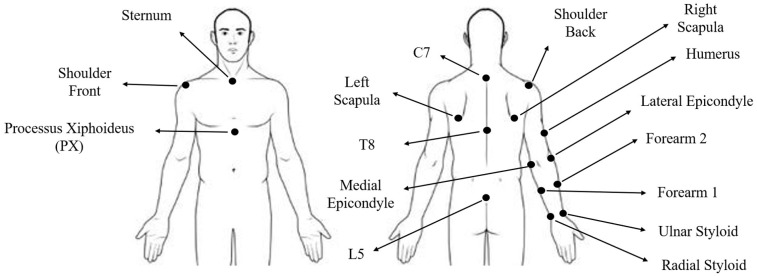
Reflective markers were attached to different locations at the trunk and right arm for kinematic monitoring of the upper body.

We used a 20-camera optical motion tracking system (Vicon Motion Systems Inc., Oxford, UK) to record the 3D kinematics of the back and the right arm. Reflective markers were attached to the participant’s skin or on the clothing, and the marker positions were tracked using Nexus (Nexus, Vicon Motion Systems Inc., Oxford, UK) with a sampling rate of 50 Hz. Markers were placed on the bony landmarks of the upper body, as shown in Figure 2, for kinematic analysis of the trunk and right arm. A total of 16 markers were affixed at their locations using double-sided tapes or Velcro straps. After the completion of the three data acquisition sessions by each participant, the marker data were postprocessed for further analysis.

### 2.3. Kinematics and Kinetics

The marker trajectories were smoothed using a 4th order low-pass Butterworth filter with a cut-off frequency of 10 Hz. We calculated the trunk inclination as the angle between the projection of the L5-C7 vertebrae vector (Figure 2) in the sagittal plane and global vertical. Moreover, to distinguish the negative angles (extension) from the positive angles (flexion), the cross-product of L5-C7 and L5-Sternum was compared against the cross-product of the L5-C7 and the global vertical. Moreover, to find the shoulder flexion, the upper arm vector was calculated from the center of front and back shoulder markers (glenohumeral joint) to the center of medial and lateral Epicondyle of elbow markers. The angle between the projection of the upper arm vector on the sagittal plane and the C7-T8 vector was calculated as the shoulder flexion.

We also calculated the moment induced by the handled box around the glenohumeral joint in the sagittal plane, as the moment is significantly larger in the sagittal plane compared with the other two orthogonal planes [31]. Towards this goal, we mapped the glenohumeral joint as well as the middle of the wrist markers at the Ulnar and Radial Styloid processes on the sagittal plane to find the projection of the shoulder-to-box vector on this plane. Next, we calculated the moment around the right glenohumeral joint, induced by half of the box weight. We further calculated and reported the cumulative moment (CM) during each loaded bending and straightening in the picking and placing as

(1) $$\mathrm{CM}=\sum_{i=1}^{n}m_{i}\delta t$$

where *n* is the number of data points during an event, $m_{i}$ is the instantaneous moment, and δt is the sampling time interval.

### 2.4. Segmentation

For a consistent investigation of the effect of repetitions on the upper-body kinematics during the palletization task, the “bending” and “straightening” occurrences were detected and compared separately with and without load. The events were detected during the first half of each session when the boxes were transported from the storage to the pallet side (palletization). The data during the second half of the session, when the process was reversed by returning the boxes to the storage side (depalletization), were discarded. As shown in Figure 1, the boxes were placed in 6 columns and 4 layers. The maximum trunk inclination occurred during the picking and placing at the ground level (first layer). Therefore, there were six picking and six placing occurrences at the ground level during the first half of each session.

**Figure 3:**
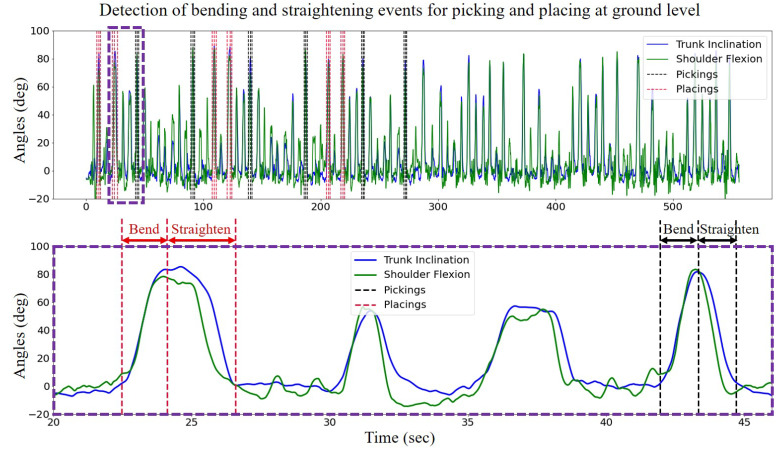
Automated segmentation of six sets of the four events at the ground level in one representative session. Every three consecutive red dashed lines mark two intervals of bending and straightening during placings, and the black dashed lines construct the bending and straightening intervals during the pickings.

For automated segmentation, we detected the lowest 24 troughs in the vertical component of the C7 marker trajectory and preserved the first 12 that occurred first, during palletization. Towards this goal, we further low-pass filtered the trajectory with a cut-off frequency of 1 Hz. Filtering at such low cut-off frequency smoothed the signal to a level that allowed the troughs to appear clearly. These troughs, which represent the maximum back flexion, mark the end of bending and the start of straightening events. Next, to find the start of bending and end of the straightening, we computed the rate of change in trunk inclination and searched it in certain proximities using a fixed-sized window before and after the maximum trunk inclination. When the rate of change exceeded a certain threshold, the point was marked as the start/end of the event. Following the detection of the start and end of all events, we time-normalized the back and shoulder flexion profiles for further analysis. The top panel in Figure 3 shows the first 12 detected events during one session. The bottom panel presents a detailed view of four representative unique events along with the trunk inclination and shoulder flexion profiles.

### 2.5. Statistical Analysis

**Figure 4:**
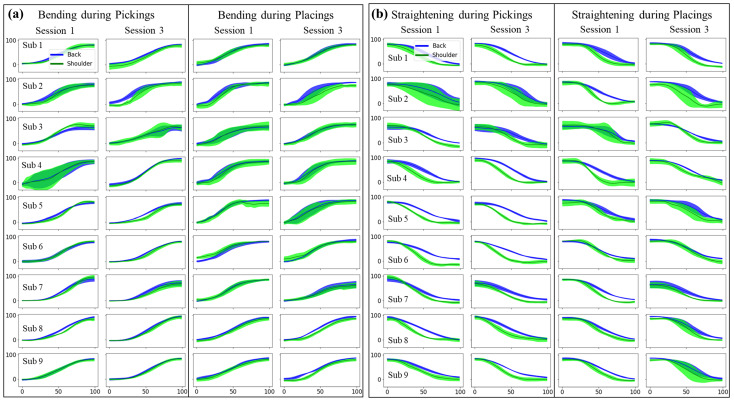
Visual comparison of the back inclination and shoulder flexion angles during (**a**) bending and (**b**) straining in the first and last sessions for the nine participants. The angles are shown with their mean and standard deviation.

We compared the detected events between the first and the third sessions across all participants. The events were compared in terms of timings, flexion and inclination angles, coordination between the back and shoulder flexion, movement variability, and the moment around the right shoulder. The timing variables included the total flexion time, time for the back/shoulder to reach their maximum, as well as 80% of maximum flexion using paired t-tests. The angles were compared in terms of maximum, mean, and median trunk inclination and shoulder flexion. Moreover, in order to investigate the coordination between the back and shoulder movements, we calculated the enclosed area between the mean of the trunk and shoulder angles (the average curves in Figure 4) and the time difference between the maximum and 80% of maximum of the trunk and shoulder angles. The movement variability was examined by exploring the standard deviation of the maximum trunk and shoulder angles, as well as mean standard deviation of the inclination/flexion angles during each event. Additionally, we compared the peak, mean, and cumulative moments around the shoulder in the sagittal plane. For statistical comparison of these parameters, we performed paired t-tests to identify the ones that demonstrated statistically significant changes between the first and the last sessions with 95% confidence (α=0.05).

## 3. Results

Figure 4 provides visual comparison of the time-normalized trunk inclination (blue line) and shoulder flexion angles (green line) of each individual participant. The curves were created by averaging six repeats of the tasks. Using our segmentation model, we detected the picking and placing events at the storage and pallet sites, respectively, and divided them into bending and straightening, creating four distinct events. Figure 4a illustrates the unloaded (without the box) bending during the picking and the loaded (with the box) bending during placing. Figure 4b further shows the loaded and unloaded straightening profiles during the picking and placing events, respectively. In Figure 4a, the back inclination during the bending events exceeds the shoulder flexion from session one to session three, and this increase is more pronounced in loaded bending during placing. This can be clearly seen in subject 2, where the back flexion in bending shows an increase over time with respect to the shoulder flexion and the increase is larger during placing as compared with the picking. Figure 4b also shows that for straightening during picking, the participants changed their movement strategy to have lower shoulder profiles with respect to the back profiles comparing sessions 1 and 3. However, straightening during placing showed the opposite trend, in general showing smaller envelops between back and shoulder profiles when sessions 1 and 3 are compared. This indicates that participants used more shoulder flexion at the end of the loaded trials.

**Figure 5:**
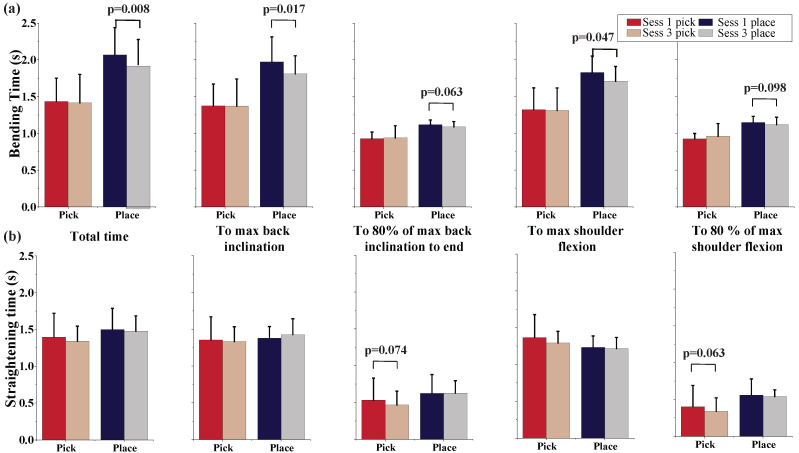
(**a**) Timings for the bending and (**b**) timings for the straightening between the first and last sessions. The bending during pickings and placings are compared separately.

For temporal parameters, Figure 5 presents the time duration associated with the back inclination and shoulder flexion during each of the four events. The bending and straightening duration during picking and placing are presented in the top and bottom panels, respectively. For each event, the total time, the time to maximum back inclination and shoulder flexion, and to 80% of the maximum back inclination and shoulder flexion are compared between the first and last sessions. During bending to reach the box, the time to maximum back inclination significantly decreased from 1.92 ± 0.32 s to 1.81 ± 0.23 s (p=0.017), and the time to maximum shoulder flexion decreased from 1.83 ± 0.21 s to 1.71 ± 0.19 s (p=0.047). No significant differences are observed in the time duration during straightening.

**Figure 6:**
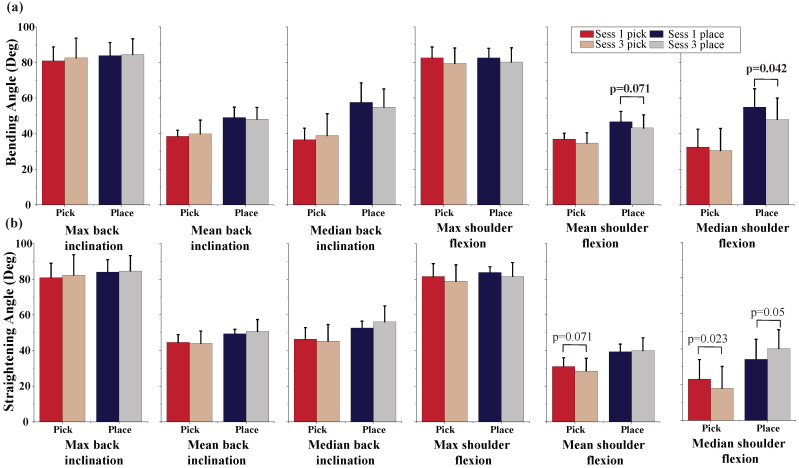
(**a**) Shoulder flexion angles during bending and (**b**) shoulder flexion angles during straightening are compared between the first and last sessions during picking and placing.

The flexion angles of back and shoulder are presented in Figure 6. The median shoulder flexion during bending in placing and straightening in picking decreased significantly from session one (54.9 ± 9.7 deg and 23.1 ± 10.3 deg) to session three (48.0 ± 11.3 deg and 17.9 ± 11.6 deg, respectively). It is noteworthy that the reduced median shoulder flexion occurred during the loaded events. We observe a statistically significant drop in the median flexion, with no significant reduction in the mean flexion. This is explained by the shoulder flexion angles that have a distribution with a peak on lower values and also containing fewer large values, which can also be phrased as a longer period spent at lower shoulder flexion followed by an abrupt transition to higher flexion angles. The shoulder flexion showed a significant decrease in the median; however, the median back inclination did not show an increasing trend. This is likely due to other kinematic factors, such as a different lower-limb posture that allows the upper limb to reach the box without bending the back. Variations in shoulder and back kinematics between the first and last sessions were also investigated during the bending and straightening events. There was no significant difference in the standard deviation of the maximum back and shoulder flexion. The variation in mean of standard deviation of the back and shoulder flexion also did not show any significant change.

**Figure 7:**
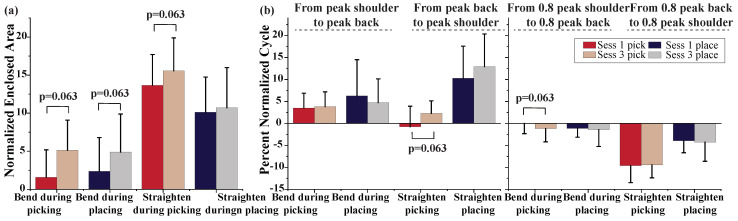
Back and shoulder coordination; (**a**) enclosed area between the mean back and shoulder flexion profiles during the four events are compared between the first and last sessions. (**b**) Percent time difference (after time normalization of profiles) between the maximum and 80% of maximum back and shoulder flexion are compared between the first and last sessions during the four events.

In Figure 7, the coordination between the shoulder and back is demonstrated by computing the normalized enclosed area between the back and shoulder angles as well as the difference of the peak angle timing. The area in Figure 7a has significantly increased from the first to the last session, except for straightening during placing: for bending during picking, it increased from 1.57 ± 3.41 to 5.13 ± 3.74; for straightening during picking, it increased from 13.6 ± 3.8 to 15.6 ± 4.1; and for bending during placing, it increased from 2.37 ± 4.17 to 4.90 ± 4.71. The enclosed area, which is a measure of coordination between the back and shoulder movements, suggests fewer coordinated back and shoulder motions. The left panel in Figure 7b shows the elapsed time from the peak shoulder flexion to the peak back inclination (after time normalization) and the right panel shows the elapsed time from 80% of maximum shoulder flexion to 80% of maximum back inclination. These variables provide a measure of back and shoulder coordination. There are no statistically significant changes between the first and last sessions in this result. From the plots, it can be observed that while in the majority of the events and sessions the right shoulder reaches its maximum flexion before the back, the back, however, reaches 80% of maximum inclination before the shoulder. This indicates shoulder flexion has a steep increase between 80% of maximum flexion and maximum flexion angles.

**Figure 8:**
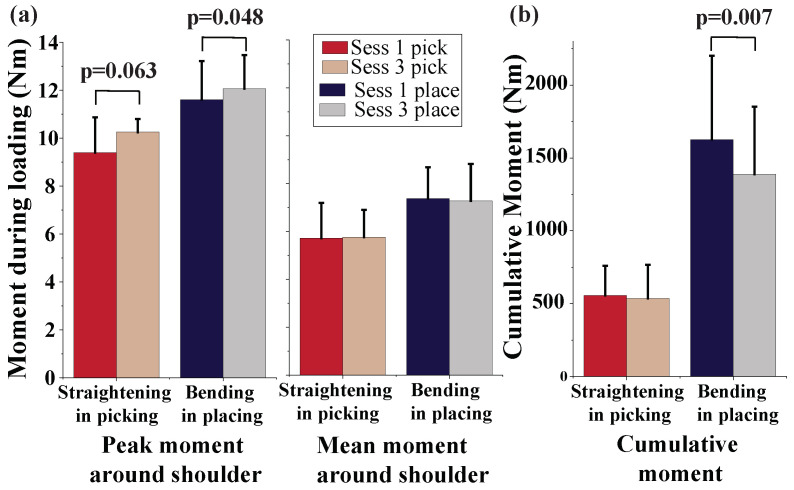
(**a**) The peak and mean moment and (**b**) the cumulative moment during the two loaded events are compared between the first and last sessions.

Kinetic analysis was also performed to measure the peak, mean, and cumulative moment around the shoulder joint in the sagittal plane for the first and last sessions. We only present the results for the two events of straightening during picking and bending during placing as we are only interested in the moment during loaded events. Statistically significant differences were observed between the peak and cumulative moments during bending in placing (Figure 8). The peak moment of shoulder is increased from 11.6 ± 1.5 Nm to 12.1 ± 1.3 Nm during placing the box (*p* = 0.048). However, the cumulative moment of shoulder is decreased from 1626 ± 543 to 1389 ± 436 Nm due to the significantly decreased time during placing of the load (*p* = 0.007).

## 4. Discussion

We investigated the effect of repetitive bending and straightening on worker biomechanics during prolonged lifting and lowering of weighted boxes in a de/palletization work setup. We focused on the warehouse and distribution centers where the order-picking workers perform repetitive palletization tasks. Nine participants completed three sessions of the simulated palletization activities. Our analysis only focuses on the picking and placing events during the work shift, and for a more detailed evaluation of the biomechanical changes, we divided each of these events into bending and straightening. The contribution of this work is to conduct a comprehensive biomechanical study investigating kinematics, task time, upper-limb coordination, and moments around the shoulder. A more comprehensive understanding of physiological changes during these tasks is crucial as fatigue or potential occupational hazards exhibit different physiological patterns for each individual [34,35].

For the investigation of the timings of each event, as compared with the percent times, absolute time provides a more meaningful indicator when biomechanical changes are assessed. Participants reduced the total bending time and the time to reach maximum back and shoulder flexion in bending during placings. It is notable that while they reached their maximum back and shoulder flexion faster in the last session (as compared with the first session) during placing, the same behavior is not observed during picking. Since the bending is loaded in placing and unloaded in picking, the weight of the box may have played an important role in the kinematic modifications adopted by the subjects. Generally, a longer time was spent on bending in placing as compared with the picking, which indicates more controlled movement during loaded bending. However, with increased repetitions, the bending time reduced significantly, which can be a potential indicator of fatigue and compensatory strategy to avoid risk of injury.

The effect of repetitive lifting on the individual’s back and shoulder kinematics has been investigated in the literature. Increased peak back flexion was reported with prolonged repetitive lifting [36,37,38]. However, different modifications of shoulder kinematics were reported in different studies. Decreased shoulder flexion, accompanied by increased elbow flexion, was observed in prolonged repetitive lifting to the chest height (to hold the box closer to the body) [39]. However, increased shoulder flexion was observed during repetitive bench-to-shoulder and overhead lifting activities [40,41]. In our study, while we observed a slight increase in the peak back inclination and a decrease in the peak shoulder flexion, the changes are not statistically significant. This could be due to the insufficient repetitions and weight of the box to induce changes in the participant’s kinematic strategy. While the mean back and shoulder flexions did not indicate a significant change between the first and the last sessions, we observed larger mean flexion during placing as compared with the picking. This translates into larger mean flexion during the loaded versus unloaded bending. However, since the increased back flexion is not compensated by a smaller shoulder flexion, it can be presumably explained by increased knee flexion during unloaded bending in picking. This can imply more ergonomic kinematics during unloaded bending (picking) as compared with the loaded bending (placing).

Additionally, repetition caused reduced coordination of the back and shoulder motions as evidenced by a significant increase in the enclosed area between the mean back and shoulder flexion. This is consistent with the reported effect of fatigue on worker kinematics in repetitive tasks [42]. Moreover, in terms of moments around the glenohumeral joint in the sagittal plane, we observed a significantly larger peak moment around the right shoulder in bending for placing. Since the peak shoulder flexion does not significantly increase in the last session during bending for placing, this may indicate a whole-body kinematic change that increased the box distance from the glenohumeral (GH) joint in the sagittal plane, which in turn lead to a larger moment arm. It should be noted that an increased shoulder flexion can only lead to a larger momentum around the GH joint if it causes the vector between the GH joint and box to make a larger angle with the gravity vector. Moreover, we observed a significant drop in the cumulative moment around shoulder. However, since a significant reduction is observed in the total bending time during placing and the mean moment around the shoulder does not indicate a significant drop, the reduction in cumulative moments can be linked to a faster bending rather than the reduced moment.

There are some limitations that can potentially reduce the generalizability of our results. First, we conducted the experiments in a laboratory setting. The controlled environment of a laboratory can reduce the generalizability of our conclusions. Therefore, a field study can better capture the variability in an uncontrolled work setting that is introduced by different heterogeneity sources. Sensors, such as inertial motion and optical fiber systems that have been developed for biomechanics applications [34,43,44,45], could be employed to support field-based measurement. Second, the duration of task performance in our study may not be sufficient to induce kinematic modifications that are observed in repetitive work settings. An extended task performance design can be beneficial to ensure the impact of repetition on task performance and preserve a longer period of kinematic alterations. Third, the study was conducted on participants who are novice to the material handling task and do not work as professional material handling workers. It would be worth exploring the difference between novice and professional material handling workers, since workers may understand the potential hazard when starting the material handling task and may thus modify their movements. Last, this study does not include lower-limb kinematics, which can be employed for future studies to capture the whole-body kinematics during the picking and placing.

This study shows important indicators that should be used to inform workers who are handling heavy materials and task designers. The joint angles are typically employed as an important indicator for monitoring work hazards, however, the time during the loaded task is also a significant factor for the workers. This study shows that worker kinematics during loaded bending could be used as an early marker for capturing the onset of fatigue and potential exposure to musculoskeletal risk. In addition, the increased peak moment around the shoulder can be also a critical indicator for monitoring potential injury. This study demonstrates that comprehensive physiological indicators, as opposed to a single metric, must be monitored and informed to workers for injury prevention. We envision that the monitoring of worker kinematics in warehousing industries, especially focusing on the loaded periods, provides significant information for early detection of kinematic changes. This will in turn allow for timely intervention to reduce musculoskeletal injuries and improvement of worker safety.

## Data Availability

Not applicable.
